# Incidence and epidemiological characteristics of congenital adrenal hyperplasia in Hangzhou newborns, China (2014–2024): a population-based cross-sectional study

**DOI:** 10.3389/fped.2026.1869920

**Published:** 2026-10-05

**Authors:** Yifei Dai, Linling Zhu, Yufeng Zeng, Yiming Chen, Xia Qian, Xiangjuan Li, Ting Wu

**Affiliations:** 1 Department of Gynecology, Hangzhou Women's Hospital, Hangzhou, Zhejiang, China; 2 The Fourth School of Clinical Medicine, Zhejiang Chinese Medical University, Hangzhou, Zhejiang, China; 3 Department of Prenatal Diagnosis and Screening Center, Hangzhou Women's Hospital (Hangzhou Maternity and Child Health Care Hospital), Hangzhou, Zhejiang, China; 4 Department of Child Healthcare, Hangzhou Women's Hospital, Hangzhou, Zhejiang, China

**Keywords:** 21-hydroxylase deficiency, birth defect, congenital adrenal hyperplasia, incidence, neonatal screening

## Abstract

**Background:**

Congenital adrenal hyperplasia (CAH) is a rare but potentially life-threatening autosomal recessive disorder. Its incidence varies by region and ethnicity. This study aimed to evaluate the birth incidence, trends, and clinical characteristics of neonatal classic CAH in Hangzhou (2014–2024) to support regional clinical prevention and management.

**Methods:**

This retrospective population-based study collected data from Hangzhou's information systems. Classic CAH cases were identified through newborn screening and clinical follow-up to age 3, with diagnosis confirmed by pediatric endocrinologists. Incidence was calculated per 100,000 live births. Categorical variables were compared using chi-square or Fisher's exact test. Temporal trend was assessed by Poisson regression.

**Results:**

Among 1,235,815 live births, 40 CAH cases were identified, giving an incidence of 3.24/100,000. The annual incidence ranged from 0/100,000 (2020) to 6.56/100,000 (2017), with no significant temporal trend (*P* = 0.297). The incidence was highest in urban (4.28/100,000), followed by suburban (2.37/100,000) and rural areas (1.93/100,000) (*P* = 0.16). Males accounted for 65% (26/40) of cases and females 35% (14/40), with a male-to-female ratio of 1.86:1. The incidence was 4.03/100,000 in males and 2.37/100,000 in females (*P* = 0.104). Among the 40 cases, salt-wasting (SW) was the predominant phenotype (33/40, 82.5%), and 21-hydroxylase deficiency accounted for 95% (38/40) of cases. Two neonates (5%) presented with salt-wasting crises, and two female infants with the simple virilizing (SV) form were initially misclassified as male. Prenatal ultrasound showed increased nuchal translucency in 6 cases (15%), adrenal enlargement in 3 cases (7.5%), and ambiguous genitalia in 1 case (2.5%). CAH was strongly associated with congenital heart defects (OR: 13.93, *P* < 0.001), and congenital hypothyroidism (OR: 42.26, *P* < 0.001).

**Conclusion:**

The incidence of classic CAH in Hangzhou (3.24/100,000) was lower than many global estimates. Although temporal and regional differences were not statistically significant, the predominance of SW cases, the occurrence of adrenal crises, and the presence of comorbidities (congenital heart defects, hypothyroidism, and cognitive developmental delay), highlight the need for improved early recognition, strengthened diagnostic capacity in suburban and rural areas, and long-term multidisciplinary management.

## 1 Introduction

Birth defects remain a major public health problem worldwide, and their incidence in China continues to be high despite improvements in prenatal screening and diagnosis (1). Congenital adrenal hyperplasia (CAH) is an autosomal recessive disease caused by defects in steroid biosynthesis (2). These defects disrupt cortisol synthesis, leading to elevated adrenocorticotropic hormone (ACTH), adrenal hyperplasia, and accumulation of steroid precursors. Affected patients typically present with glucocorticoid and mineralocorticoid deficiencies, accompanied by symptoms of hyperandrogenism.

The most common form, 21-hydroxylase deficiency (21-OHD), accounts for 90%–95% of all cases and is caused by mutations in the CYP21A2 gene on chromosome 6p21.3. CAH is classified into classic and non-classic forms; the classic form includes simple virilizing (SV, ∼25% of cases) and salt-wasting (SW, ∼75% of individuals) phenotypes (3). In the SW form, both cortisol and aldosterone are deficient; without prompt treatment, affected infants can develop life-threatening adrenal crises characterized by electrolyte imbalances, hypovolemia, and shock. The SV form retains about 1%–2% residual enzyme activity and causes varying degrees of androgen excess, leading to ambiguous genitalia in newborn girls, early puberty in children (4), and short adult height (5).

For the classic form, global incidence estimates range from 1 in 10,000 to 1 in 20,000 live births (2). A recent multi-country study (16 countries, published since 2008) reported rates between 1:14,000 and 1:18,000 (6). Rates vary by ethnicity and geography. For example, Italy reported 1:17,699 (7), India 1:5,762 (8), and Japan 1:19,859 (9). For China, a meta-analysis reported an overall incidence of roughly 1:23,024 (10), which is lower than in most Western countries. Even so, epidemiological data on CAH from mainland China are still scarce.

Over the past decade, Hangzhou, a major city in eastern China with a population of over 12 million, has undergone rapid urbanization and healthcare development. Understanding the local disease burden is essential for designing region-specific prevention strategies, optimizing healthcare resource allocation, and improving outcomes for affected children. However, estimating the population-based incidence of classic CAH presents several challenges. CAH is rare, requiring large birth cohorts for stable estimates; case identification depends on screening program performance, which may vary over time and across regions; and early neonatal deaths or misdiagnosis may lead to under-ascertainment. These challenges highlight the need for standardized surveillance. Therefore, this study aimed to assess the incidence, temporal trends, and epidemiological characteristics of classic CAH in Hangzhou to inform clinical prevention and treatment decisions. To our knowledge, it is the first population-based study to systematically examine these aspects in this region using an 11-year birth cohort of over 1.24 million newborns. The findings are intended to inform clinical prevention, early diagnosis, and treatment strategies, and to provide evidence for strengthening newborn screening programs and healthcare resource allocation in Hangzhou and similar settings in China.

## 2 Materials and method

### 2.1 Data sources and quality control

This retrospective population-based study used data from the Hangzhou Maternal Child Health Information System, the Hangzhou Delivery Information Management System, and the Zhejiang Newborn Disease Screening Information Management System. These systems record parental sociodemographics, maternal prenatal examination details, newborn birth and screening results, birth defect monitoring data, and health follow-up for children up to 3 years of age. All hospitals in Zhejiang Province offering prenatal and delivery services are required by policy to register and upload maternal and newborn information to these systems. In parallel, community physicians are responsible for entering child health follow-up data. Since 2014, Zhejiang Province has offered partially subsidized newborn screening to all infants born in regulated medical facilities, covering CAH, congenital hypothyroidism (CH), phenylketonuria, and glucose-6-phosphate dehydrogenase (G6PD) deficiency. The database used a restricted online sharing scheme. Dedicated staff at Hangzhou Women's Hospital reviewed and verified all information. Quality control checks were performed monthly at the district level and semi-annually at the city or provincial level to ensure complete case report forms, accuracy of diagnoses, and minimization of underreporting.

The study included 1,235,815 pregnant women and their newborns across 13 districts, counties, and cities in Hangzhou, from January 2014 to December 2024. Fetuses affected by birth defects that resulted in abortion, termination, or stillbirth were excluded. The Ethics Committee of Hangzhou Women's Hospital approved the study [(2021) Medical Ethics Review A (7–13)], and all data were anonymized before analysis.

### 2.2 Case ascertainment and diagnostic criteria

The complete CAH screening and diagnostic pathway is summarized in Figure 1. Briefly, cases were identified through two complementary pathways. The first was the newborn disease screening system. Routine blood sampling was performed after 48 h of life and within the first 7 days, after adequate feeding. For infants who missed the initial screening (due to prematurity, low birth weight, ongoing illness, early discharge, or other reasons), the sampling window was extended, generally to no later than 20 days after birth. Screening used a 17-hydroxyprogesterone (17-OHP) cut-off of 11.5 nmol/L, with a higher recall threshold of 32 nmol/L for preterm or low-birth-weight infants. Urgent recall was triggered at >25 nmol/L for full-term and >50 nmol/L for preterm infants. At recall, 17-OHP was re-measured using liquid chromatography-tandem mass spectrometry (LC-MS/MS), along with biochemical tests (including blood electrolytes and other steroid profiling) and karyotype analysis. Genetic testing was performed when biochemical results were inconclusive.

**Figure 1:**
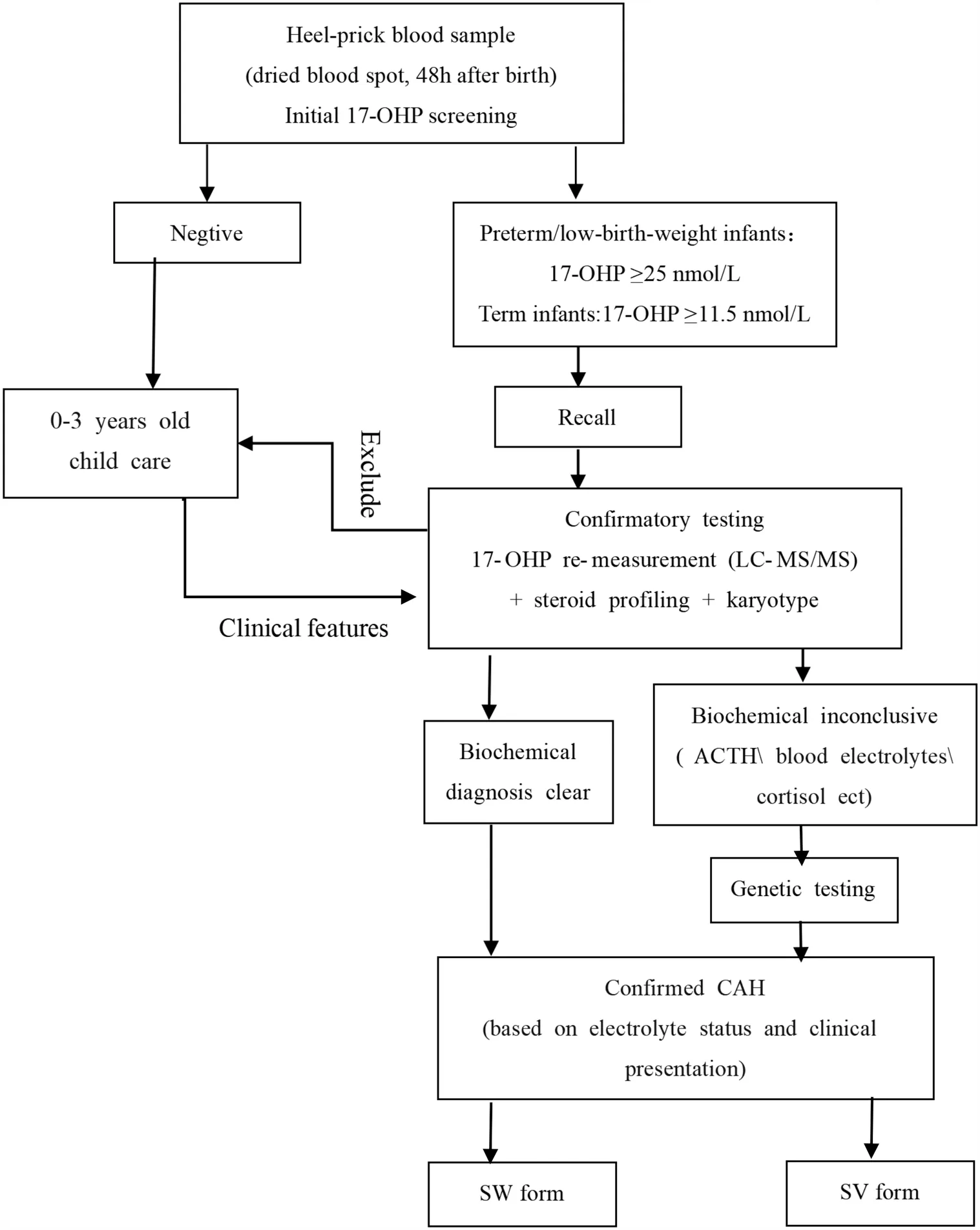
CAH screening and diagnostic pathway in Hangzhou. LBW: low birth weight; LC-MS/MS: liquid chromatography-tandem mass spectrometry; 17-OHP: 17-hydroxyprogesterone; ACTH: adrenocorticotropic hormone; SW: salt-wasting; SV: simple virilizing..

The second pathway involved the Hangzhou Maternal Child Health Information System, which followed children up to 3 years of age. The system captured infants who presented with clinical features suggestive of CAH, such as ambiguous genitalia, salt-wasting crises, or precocious puberty, but had not been detected by newborn screening.

Diagnosis followed the Chinese consensus (11) and was confirmed by pediatric endocrinologists or metabolism specialists based on the following criteria: (a) persistently elevated blood 17-OHP; (b) elevated androstenedione and testosterone; (c) elevated or normal ACTH; (d) in SW forms, low sodium and high potassium; (e) pathogenic variants identified by genetic testing; (f) clinical manifestations, including hyperpigmentation and, in females, clitoral enlargement. A diagnosis could be established by meeting criteria (a), (b), and (c) simultaneously, or criteria (a) and (e) together.

Clinical phenotyping, distinguishing SW from SV forms, was based on the presence of hyponatremia, hyperkalemia, or salt-wasting crises requiring intravenous fluid and mineralocorticoid replacement (SW), versus the absence of such findings with only androgen excess features (SV). Non-classic CAH was not captured because it typically presents later in life with milder symptoms and is not identified by newborn screening or routine early-childhood surveillance.

Subtype classification based on enzyme deficiency was performed by integrating biochemical, steroid metabolic, and (when available) genetic data. 21-OHD was identified by markedly elevated 17-OHP, androstenedione, and testosterone. For 11*β*-hydroxylase deficiency (11*β*-OHD), the key features were elevated 11-deoxycortisol and deoxycorticosterone, often accompanied by hypertension and hypokalemia, which distinguishes it from 21-OHD. 3*β*-hydroxysteroid dehydrogenase deficiency (3*β*-HSD2) was characterized by elevated 17-OHP and dehydroepiandrosterone, with an increased 17-OHP/cortisol ratio. Genetic testing for CYP21A2, CYP11B1, and HSD3B2 was performed in selected cases, particularly when biochemical profiles were equivocal. For cases without genetic confirmation, subtype assignment was made by pediatric endocrinologists based on comprehensive clinical and biochemical evaluation, following the Chinese consensus (12).

CH screening used the same dried blood spot samples. Thyroid Stimulating Hormone (TSH) was measured by time-resolved fluoroimmunoassay. The initial recall threshold was 8 *μ*IU/mL; infants with TSH ≥15 μIU/mL were urgently recalled for confirmatory testing, which included serum TSH and free thyroxine (FT4) measurements. A pediatric endocrinologist confirmed CH when serum TSH was elevated and FT4 was below the age-specific reference range.

### 2.3 Variables and definitions

We extracted maternal characteristics including age at delivery (<35, ≥35 years), education level, and pregnancy complications (hypertensive disorders, gestational diabetes, abnormal liver function, anemia and polyhydramnios). Newborn characteristics included gender, residential area (urban, suburban, rural), gestational age, birth weight, birth length, prenatal ultrasound findings, congenital congential disorders [e.g., ambiguous genitalia, congenital heart defects (CHD), hypothyroidism], and neonatal complications (e.g., neonatal jaundice). Prenatal ultrasound findings include nuchal translucency (NT) thickness at 11–13⁺⁶ weeks (normal: <2.5 mm; increased: ≥2.5 mm), enlarged adrenal glands on second- or third-trimester ultrasound, and ambiguous genitalia. Follow-up data include growth and development parameters up to 3 years of age, as well as assessments of cognitive development.

Residential areas were classified according to postal codes and administrative divisions under the General Planning of Land Space of Hangzhou (2021–2035): urban (Shangcheng District, Xihu District, Gongshu District, Binjiang District), suburban areas (Xiaoshan District, Yuhang District, Linping District, Qiantang District, Fuyang District, Lin'an District), and rural (Jiande City, Tonglu County, Chun'an County).

### 2.4 Statistical analysis

Incidence of classic CAH was calculated as the number of confirmed cases divided by total live births, expressed per 100,000 live births. Data were managed using Excel 2013, and all statistical analyzes were performed with IBM SPSS Statistics version 30.0. For categorical variables, comparisons were made using the chi-square test or Fisher's exact test, as appropriate. Poisson regression was used to analyze the temporal trend of CAH incidence, with the natural logarithm of the annual number of live births included as an offset. All tests were two-sided, and a *P* value < 0.05 was considered statistically significant. There were no missing data for the variables included in the analysis, as the information systems required complete reporting, and regular quality control audits confirmed data completeness.

Birth weight and length were converted to standard deviation scores (SDS) based on the Chinese neonatal growth reference for gestational age and sex (13). Since the reference provides percentiles rather than means and standard deviations, the 50th percentile (P50) was used as an approximation of the population mean. We estimated the standard deviation (SD) using the formula SD ≈ (P90 - P50)/1.28, as previously described (14). SDS was then calculated as (measured value - P50)/the estimated SD.

## 3 Results

### 3.1 Overall incidence and temporal trends

During 2014–2024, Hangzhou recorded 1,235,815 live births and 40 classic CAH cases, yielding an incidence of 3.24 per 100,000 live births (1 in 30,864). Annual incidence ranged from 0 per 100,000 (2020) to 6.56 (2017) per 100,000, with higher rates also seen in 2017 (6.56), 2022 (4.50) and 2023 (5.56) (Table 1, Figure 2). Poisson regression analysis showed no statistically significant change over the study period [annual percent change (APC) = 5.4%, 95% confidence interval (CI): −4.5% to 16.3%, *P* = 0.297].

**Table 1:** Incidence of classic CAH in Hangzhou, 2014−2024.

Year	The number of live births (n)	CAH (n)	Incidence (/ 100,000)	95%CI (/100,000)
2014	131,128	2	1.53	0.18–4.80
2015	102,734	3	2.92	0.60–7.55
2016	141,528	3	2.12	0.44–5.48
2017	137,184	9	6.56	3.00–12.45
2018	121,447	3	2.47	0.51–6.39
2019	122,934	4	3.25	0.89–7.45
2020	101,003	0	0	0–2.49
2021	95,876	3	3.13	0.65–8.09
2022	88,888	4	4.50	1.23–10.30
2023	89,862	5	5.56	1.81–11.70
2024	103,231	4	3.87	1.06–8.87
Total	12,35,815	40	3.24	2.31–4.40

CAH, congenital adrenal hyperplasia; CI, confidence interval.

**Figure 2:**
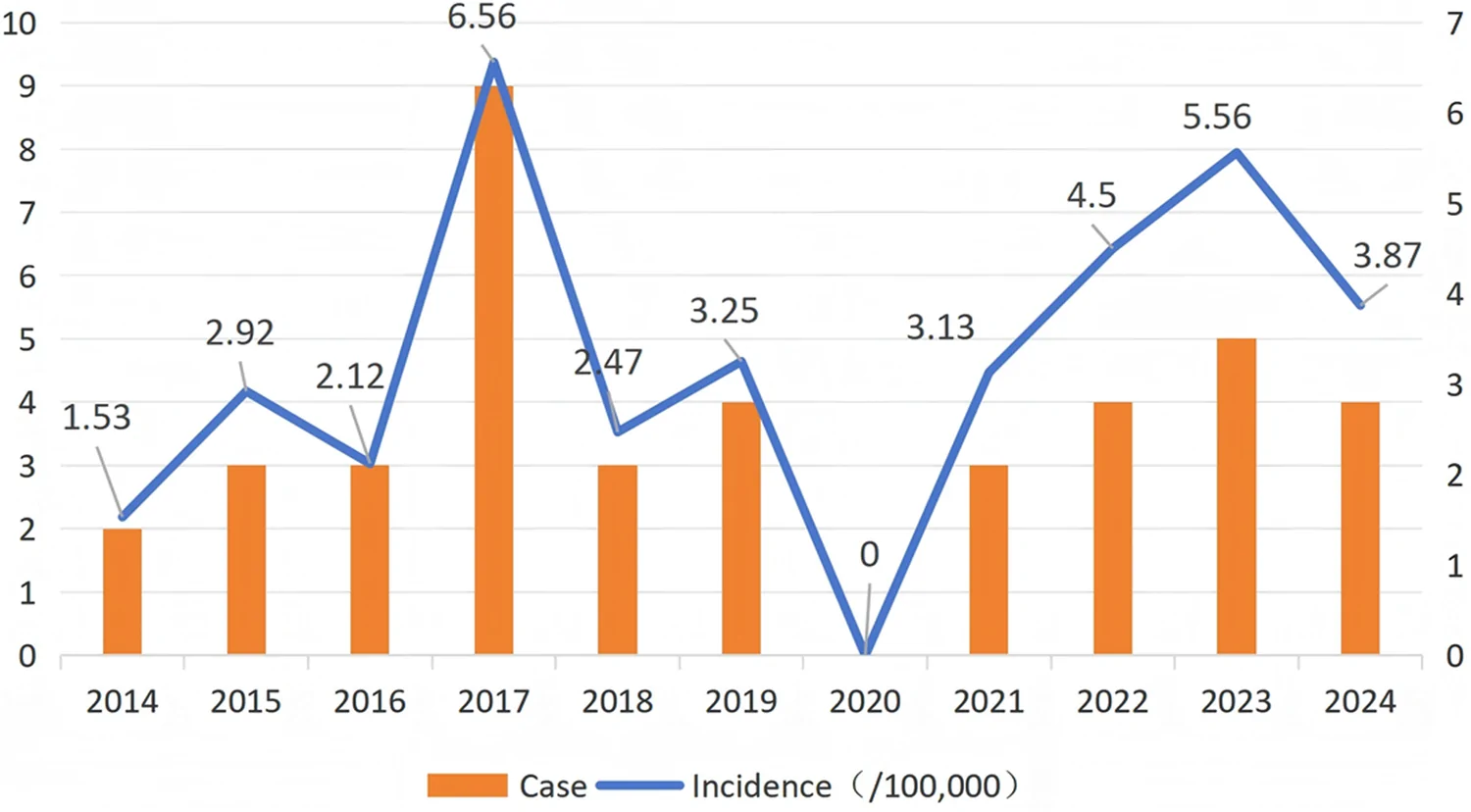
Incidence and trends of neonatal classic CAH in Hangzhou, 2014−2024

#### 3.1.1 Mode of diagnosis

The majority of cases (31/40, 77.5%) were identified through newborn screening and were asymptomatic at diagnosis. The remaining 9 cases (22.5%) were diagnosed after clinical presentation. All screening-identified cases were diagnosed within the first 2 months of life. Among clinically diagnosed cases, age at diagnosis ranged from 5 days to 8 months. Detailed data on age at diagnosis and presenting symptoms were not consistently available for all clinically diagnosed cases from the community health records, which limited our ability to provide a complete symptom profile.

#### 3.1.2 Regional differences

Incidence was highest in urban areas (4.28 per 100,000), followed by suburban (2.37) and rural areas (1.93). Logistic regression showed no significant association between residential area and CAH risk (*P* = 0.16) (Table 2).

**Table 2:** Clinical characteristics of newborns with classic CAH in Hangzhou, 2014–2024.

Characteristic	Frequency	Percentage (%)
Gender		
Male	26	65
Female	14	35
Maternal residence		
Urban areas	25	62.5
Suburban areas	13	32.50
Rural areas	2	5.00
Twin gestation		
Yes	1	2.5
No	39	97.5
Preterm birth		
Yes	5	12.5
No	35	87.5
Classic CAH type		
21-OHD	38	95
11*β*-OHD	1	2.5
3β-HSD2	1	2.5
Clinical phenotype		
SW forms*	33	82.5
SV forms	7	17.5
Family history of CAH		
Yes	1	2.5
No	39	97.5
NT ≥ 2.5mm		
Yes	6	15
No	34	85
Prenatal enlarged adrenals		
Yes	3	7.5
No	37	92.5
Prenatal ambiguous genitalia		
Yes	1	2.5
No	39	97.5
Neonatal jaundice		
Yes	6	15
No	34	85
CHD		
Yes	5	12.5
No	35	87.5
CH		
Yes	2	5
No	38	95
Growth retardation		
Yes	1	2.5
No	39	97.5
Cognitive developmental delay		
Yes	1	2.5
No	39	97.5

*SW includes 31 cases of 21-OHD, 1 case of 11β-OHD, and 1 case of 3β-HSD2 (all presenting with salt-wasting crises or electrolyte abnormalities requiring mineralocorticoid replacement).

CAH, congenital adrenal hyperplasia; 21-OHD, 21-hydroxylase deficiency; SV, simple virilizing; SW, salt-wasting; 11β-OHD, 11β-hydroxylase deficiency;3β-HSD2, 3β-hydroxysteroid dehydrogenase deficiency; NT, Prenatal nuchal translucency; CH, congenital hypothyroidism; CHD, Congenital heart defects.

#### 3.1.3 Sex distribution

Of the 40 cases, 26 (65%) were male and 14 (35%) females, giving a male-to-female ratio of 1.86:1. The incidence was 4.03 per 100,000 in males and 2.37 per 100,000 in females; the difference was not statistically significant (*P* = 0.104) (Table 3).

**Table 3:** Sex-specific incidence of classic CAH in Hangzhou, 2014–2024.

Gender	Number of births (n)	Classic CAH cases (n*,* %)	Incidence (1/100,000)	*χ* ^2^	P
Male	6,44,552	26 (65%)	4.03	2.644	0.104
Female	5,91,263	14 (35%)	2.37		

CAH, congenital adrenal hyperplasia.

### 3.2 Clinical presentation and diagnostic delays

#### 3.2.1 Maternal characteristics

Among the 40 mothers, 35 (87.5%) were under 35 years old, and 29 (72.5%) held an associate or bachelor's degree. One pregnancy (2.5%) was achieved through assisted reproductive technology. Pregnancy complications were uncommon: 2 mothers (5%) had hypertensive disorders of pregnancy (HDP) requiring medication, one (2.5%) had gestational diabetes mellitus (GDM), and 4 (10%) showed abnormal liver function. Anemia and polyhydramnios each occurred in 2 pregnancies (5%) (Supplementary Table S1).

#### 3.2.2 Clinical characteristics of children with CAH

Among the 40 CAH cases, 38 (95.0%) were 21-OHD, one (2.5%) was 11*β*-OHD, and one (2.5%) was 3*β*-HSD2. When classified by clinical phenotype, 33 cases (82.5%) presented with SW features (including the single 11*β*-OHD and 3*β*-HSD2 cases), while the remaining 7 cases (17.5%) were SV type. A positive family history of CAH was reported in one patient (2.5%) (Table 2).

#### 3.2.3 Adrenal crises and diagnostic delays

Two neonates (5.0%) presented with salt-wasting crises, one on day 5 and the other on day 7 of life, both requiring emergency intervention with intravenous fluids and mineralocorticoids. One additional infant had documented hypoglycemia, and another had hypokalemia at presentation. Notably, one infant with SW form was initially managed as suspected sepsis before the correct diagnosis was established. Two infants with the SV form were initially misidentified as male at birth and later underwent genital reconstructive surgery (at 4 and 10 months of age).

#### 3.2.4 Prenatal ultrasound findings

Prenatal ultrasound records showed increased NT in 6 cases (15.0%), enlarged fetal adrenal glands in 3 cases (7.5%), and ambiguous genitalia in 1 case (2.5%). One case (2.5%) had both increased NT and enlarged adrenal glands.

#### 3.2.5 Treatment and follow-up

All 40 patients initiated glucocorticoid therapy within the first 4 months of life; The parents of one patient stopped medication against medical advice. Through the Child Health Information System, patients were followed until 3 years of age. Three children had mild early growth delay; two had caught up by 6 months, while one remained slightly behind. One child with the SW form, who had no history of birth asphyxia or resuscitation, was diagnosed with intellectual developmental delay. All patients were alive at the end of follow-up.

### 3.3 Associated comorbidities and birth size

#### 3.3.1 CHD and other comorbidities

Among the 40 CAH patients, 5 (12.5%) had CHD, compared with 12,541 (1.01%) non-CAH live births during the same period (OR = 13.93, 95% CI: 5.46–35.57, *P* < 0.001). Two patients (5.0%) had congenital hypothyroidism, compared with 1,537 (0.12%) non-CAH live births [odds ratio (OR) = 42.26, 95% CI: 10.19–175.34, *P* < 0.001]. In addition, neonatal jaundice occurred in 6 (15.0%), growth retardation in one (2.5%), and cognitive developmental delay in one (2.5%) (Table 4).

**Table 4:** Association between classic CAH, CHD and CH.

Group	Classic CAH		OR (95%CI)	P
	Yes (n)	No (n)		
CHD				
Yes	5	12,541	13.93 (5.458–35.572)	<0.001
No	35	1,223,234	Ref	
CH				
Yes	2	1,537	42.26 (10.187–175.34)	<0.001
No	38	1,234,238	Ref	

CAH, congenital adrenal hyperplasia; CHD, Congenital heart defects; CH, congenital hypothyroidism; OR, odds ratio; CI, confidence interval.

#### 3.3.2 Birth weight and length

The mean birth weight was 3,254.25 ± 585.82 g, corresponding to a mean birth weight SDS of 0.34 ± 1.12 relative to the Chinese neonatal growth reference for gestational age and sex (13). The mean birth length was 49.08 ± 2.13 cm, with a mean SDS of −0.13 ± 0.72 (Table 5). These SDS values fell within the normal range for the reference population, indicating that the birth weight and length of CAH infants in our cohort were comparable to those of the general population of the same gestational age and sex.

**Table 5:** Birth weight and length of newborns with classic CAH.

Variable	All (*n* = 40)	Male (*n* = 26)	Female (*n* = 14)
Birth weight (g)	3,254.25 ± 585.82	3,281.92 ± 526.17	3,202.86 ± 701.98
Birth weight (SDS)	0.34 ± 1.12	0.29 ± 1.12	0.44 ± 1.15
Birth length (cm)	49.08 ± 2.13	49.46 ± 1.50	48.36 ± 2.90
Birth length (SDS)	−0.13 ± 0.72	−0.08 ± 0.61	−0.22 ± 0.92

The data are expressed as mean ± SD.

SDS, standard deviation scores; SD, standard deviation.

## 4 Discussion

This population-based study provides a comprehensive epidemiological overview of classic CAH in Hangzhou from 2014 to 2024. To our knowledge, it is the first study to systematically examine the incidence, trends, and clinical characteristics of classic CAH in this region using a large, population-based birth cohort.

### 4.1 Incidence and case ascertainment

The overall incidence of classic CAH in our cohort was 3.24 per 100,000 live births (approximately 1 in 30,864 live births), which is lower than most global estimates for classic CAH (ranging from 1 in 10,000 to 1 in 20,000 live births) and also below figures reported from other Chinese regions and Asian populations, such as Japan 1:19,859 (9), Taiwan 1:14,822 (15), Fujian 1:26,883 (16), Guangzhou 1:16,866 (17).

This difference likely has several explanations. First, genetic variation across populations is well recognized in CAH, with carrier frequencies and mutation spectra varying by ethnic groups (18). The carrier rate in Zhejiang Province may be lower than in some other regions, leading to a genuinely lower incidence. Furthermore, Hangzhou has a large migrant population from other provinces. This admixture may have increased genetic diversity, which could lower the observed carrier frequency of CYP21A2 pathogenic variants. Consequently, the incidence we report may be lower than that of a more geographically and genetically isolated population. Second, our surveillance system extended only to 3 years of age and relied on newborn screening and early childhood clinical follow-up. In Zhejiang, recall for positive screening results is typically scheduled 7–10 days after birth. However, salt-wasting crises can develop within the first few days of life, before screening results are available. Affected infants may present with non-specific signs such as poor feeding, vomiting, dehydration, or shock, and may be misdiagnosed as neonatal sepsis. In our cohort, one infant with SW form was initially managed as suspected sepsis before the correct diagnosis was established. Moreover, our surveillance database was not linked to mortality records. We therefore cannot exclude the possibility that some infants who died before screening, or whose deaths were attributed to other causes, were not captured. If such under-recognition occurred, the true incidence of classic CAH in Hangzhou could be higher than our estimate suggests. Third, variations in screening protocols, diagnostic criteria, and reporting practices across regions may contribute to observed differences (19).

The observed temporal fluctuations, including higher rates in 2017 and 2023, are likely influenced by policy changes and improvements in diagnostic methods rather than a genuine change in disease occurrence. The introduction of a free universal screening program for birth defects (including CAH) in January 2016 probably increased screening coverage and detection rates. This could explain the first incidence peak in 2017 (6.56 per 100,000). In addition, the integration of the birth defect monitoring system into the community health management network in October 2020, together with the gradual recovery of screening and reporting systems after the COVID-19 pandemic, may have contributed to the second incidence peak observed in 2023. However, the small number of annual cases (often fewer than 5) also makes it difficult to detect a true trend; random variation alone could produce the observed peaks and troughs. Overall, there is no strong evidence of a sustained increase or decrease over the 11-year period.

Regional differences were also observed: incidence of classic CAH was higher in urban areas (4.28/100,000) than in suburban (2.37/100,000) or rural areas (1.93/100,000), though these differences were not statistically significant (*P* = 0.16). This pattern likely reflects disparities in healthcare access and diagnostic capacity rather than true biological differences. Urban districts have more tertiary hospitals with specialized pediatric endocrinology services, advanced laboratories, and well-established community health monitoring systems, which may lead to more complete case ascertainment. Conversely, suburban and rural districts have more migrant residents, who may be less likely to respond to screening recall or complete follow-up, and healthcare providers in these regions may be less familiar with CAH. Similar geographic disparities have been reported for other congenital anomalies in China, including hypospadias (20).

Regarding sex distribution, the male-to-female ratio in our cohort was 1.86:1 (65% male, 35% female), which deviated from the expected 1:1 ratio for an autosomal recessive disorder, although the difference was not statistically significant (*P* = 0.104). This male predominance has been observed in other Chinese studies (10) and may reflect several factors: parents of male infants may seek medical advice more often; China has a known male-skewed sex ratio at birth (about 1.10:1); and newborn screening may be less sensitive in detecting affected females due to sex-based differences in 17-OHP levels (21).

### 4.2 Clinical presentation and diagnostic delays

The salt-wasting form accounted for 82.5% of CAH cases in our cohort (33/40, including the single 11*β*-OHD and 3*β*-HSD2 cases), similar to the reported proportion (22). This may partly reflect preferential detection of more severe phenotypes, as our surveillance network is likely more sensitive to cases presenting early with overt symptoms. SV forms, particularly in males, often do not become clinically apparent until later childhood (e.g., with premature adrenarche, rapid growth, or other signs of androgen excess) and may therefore be under-represented within the 3-year follow-up window. Newborn screening algorithms may also have lower sensitivity for SV forms, which typically show milder 17-OHP elevations.

Although deaths due to adrenal crises have become rare since the implementation of newborn screening programs (23), adrenal crises remain a potentially life-threatening condition in patients with CAH. Approximately one-fifth of children with classic CAH experience an adrenal crisis during the first four years of life (24). The two neonates (5%) presented with salt-wasting crises in the first week, and one infant was initially misdiagnosed with sepsis. In Zhejiang, recall for positive screening results is typically scheduled 7–10 days after birth, but salt-wasting crises can develop within the first few days of life, before screening results are available. This highlights a critical gap: the current recall interval may be too long for infants at risk of life-threatening adrenal crises. These findings suggest that medical personnel should be trained to recognize early signs of adrenal crises and to start treatment quickly (25). Strategies to shorten this interval, or to provide emergency guidance to parents before recall, could be beneficial.

#### 4.2.1 Prenatal ultrasound findings

We found that 15% of infants with CAH had increased NT (≥2.5 mm) and 7.5% had fetal adrenal hyperplasia—both higher than the background population. Increased NT is a well-established marker for chromosomal abnormalities and structural anomalies (26, 27). Moreover, several case reports have described an association between increased NT thickness and CAH (28, 29), suggesting that this ultrasound sign could be an early prenatal indicator. The mechanism may involve cortisol deficiency, which creates an abnormal hormonal environment during fetal life. Cortisol deficiency could affect lymphatic development (30), alter extracellular matrix composition (31), or directly cause generalized edema.

Fetal adrenal enlargement in CAH has also been described in several case reports (32, 33). In fetuses with CAH, high ACTH levels stimulate the adrenal glands, causing hyperplasia that can be seen on ultrasound as early as the second trimester. However, the sensitivity of adrenal enlargement as a marker for CAH requires further investigation. Adrenal enlargement may not present in all affected newborns; one report found only 2 out of 25 patients exhibiting this finding (33). Similarly, we recognized only 3 out of 40 newborns with CAH had enlarged adrenals. Nevertheless, longitudinal adrenal measurements have been proposed as a potential tool to monitor affected female fetuses receiving corticosteroid therapy. Adrenal imaging also plays an important role when biochemical diagnosis of CAH is difficult, as well as during follow-up and pre-surgical evaluation (34).

The single case with prenatally detected ambiguous genitalia (2.5%) is likely an underestimate. Detailed fetal anatomy surveys do not always include thorough evaluation of external genitalia, and subtle abnormalities can be missed. In female fetuses with CAH, virilization of the external genitalia can be detected on ultrasound from around 18–20 weeks gestation (35). Moreover, China's law prohibits non-medical sex determination, which may discourage detailed assessment of fetal genitalia during routine scans. Some abnormally virilized genitalia may simply be regarded as male, and affected females could go unrecognized prenatally, further contributing to underdetection.

These findings suggest that prenatal ultrasound can help identify fetuses at risk for CAH. While NT thickening and adrenal enlargement are non-specific findings, their presence, particularly in combination, should prompt careful evaluation of fetal genitalia, as well as genetic counseling and prenatal diagnosis in appropriate cases. For a fetus with NT thickening or adrenal enlargement, a normal karyotype, and no other obvious structural anomalies, clinicians should remain vigilant for CAH, especially in the presence of a family history. Future large-scale prospective studies are needed to further quantify the strength of this association and elucidate the underlying pathophysiological mechanisms.

### 4.3 Clinical and public health implications

The burden of CAH extends beyond incidence to include associated comorbidities that affect long-term health and development. In our CAH cohort, 12.5% had CHD, much higher than in the general population (OR 13.92, *P* < 0.001). Similarly, 5.0% had CH, with an OR of 42.26 (*P* < 0.001). This matches previous reports of an increased risk of cardiac anomalies or CH in CAH patients (36–38). The underlying mechanisms remain unclear. One possibility is that both conditions arise from shared genetic or developmental pathways. A case report (39) described a non-consanguineous family where four siblings had both CAH and CH, suggesting that the two conditions can co-occur by chance as independent autosomal recessive disorders. Given our small sample size, the observed association may be incidental, but it warrants attention in clinical practice.

We also observed that birth weight and length SDS values in our cohort fell within the normal range (weight SDS +0.34; length SDS −0.13), indicating that birth size was comparable to that of the general population. This is consistent with some previous reports suggesting that prenatal androgen exposure may not substantially affect birth size (40). In contrast, other studies found larger birth size in CAH infants (41, 42). When analyzed by sex, female infants showed a slightly higher mean birth weight SDS (+0.44) than males (+0.29), and slightly shorter length SDS (−0.22) than males (−0.08). The slightly higher birth weight SDS in females may be related to the anabolic effects of prenatal androgen excess, which some suggest may influence fetal weight gain (43). These discrepancies across studies may reflect differences in study populations, sample sizes, or genetic backgrounds. Further research with larger cohorts and standardized growth references is needed to clarify the relationship between CAH, androgen exposure, and fetal growth parameters.

Early childhood follow-up identified one child (2.5%) with cognitive developmental delay. This child had no history of hypoglycemia or salt-wasting crises, factors associated with neurodevelopmental impairment in other studies (32, 33). However, chronic glucocorticoid exposure can alter brain structure (44), and accompanying metabolic disturbances may affect cognitive function (45). Yet-unidentified genetic modifiers may also play a role. This observation reinforces the need for long-term neurodevelopmental monitoring.

These findings have several practical implications for resource allocation and public health policy. First, the geographic disparities in CAH incidence probably reflect differences in case identification, though not statistically significant. Rates were higher in urban areas (4.28/100,000) than in suburban (2.37/100,000) and rural (1.93/100,000) areas, highlighting the need to strengthen diagnostic capacity in suburban and rural areas, and to prioritize these efforts to reduce potential underdiagnosis. Second, SW was the predominant phenotype in our cohort (82.5%) and two neonates developed adrenal crises in the first week of life. These findings highlight the need for training front-line providers to recognize early signs of adrenal crisis, such as poor feeding, vomiting, dehydration, or shock. Earlier recognition and prompt intervention could reduce diagnostic delays and prevent fatalities. Third, the maternal characteristics in our cohort (most mothers were under 35 years, had at least an associate degree) suggest that antenatal education about CAH could effectively reach this population through existing maternity and primary healthcare channels. Fourth, the associations with CHD and CH support routine cardiac and thyroid evaluation in infants diagnosed with CAH, and the finding of cognitive developmental delay reinforces the need for long-term multidisciplinary follow-up.

Taken together, efforts to improve outcomes for children with CAH in Hangzhou should focus on: (1) strengthening diagnostic capacity in suburban and rural areas; (2) training front-line healthcare professionals in the recognition of adrenal crises; (3) developing clear referral pathways to specialist pediatric endocrinology services; and (4) ensuring long-term multidisciplinary follow-up for affected children.

## 5 Study strengths and limitations

This study has several strengths. It is a large, population-based study covering over 1.24 million births across 11 years, with standardized surveillance systems and regular quality control. The inclusion of prenatal ultrasound findings also offers unique insights into potential early markers of CAH.

However, several limitations should be acknowledged. First, our follow-up was limited to 3 years of age, so our incidence estimates cover only classic CAH detected through newborn screening and early childhood surveillance. Late-presenting cases, particularly SV forms in males, were not captured. As a result, our case ascertainment may be skewed toward more severe phenotypes. Second, our database was not linked to mortality records, so we could not ascertain whether any CAH-related deaths occurred before diagnosis or were misclassified as other causes (e.g., neonatal sepsis). Third, the retrospective design and lack of a healthy control group limited our ability to analyzes risk factors for CAH. Fourth, detailed data on genotype-phenotype correlations, parental consanguinity, family history and parental geographical origin were not available, limiting our exploration of genetic and inheritance patterns. Finally, detailed information on age at diagnosis, presenting symptoms, and the time interval from symptom onset to treatment initiation was not consistently recorded in the community health records for all clinically diagnosed cases. This limited our ability to provide a complete symptom profile or to systematically analyze factors associated with diagnostic delay.

Future research should focus on prospective multicenter studies with standardized protocols and healthy control groups to confirm our findings and enable robust risk factor analysis. Establishing national or regional CAH registries would facilitate genotype-phenotype correlation studies and the assessment of long-term outcomes. Additionally, systematic assessment of prenatal ultrasound markers (e.g., adrenal size) in at-risk pregnancies is needed to determine their sensitivity and specificity for early CAH detection. The observed associations with CHD, CH, and neurodevelopmental outcomes require confirmation in larger cohorts, along with investigation into underlying mechanisms.

## 6 Conclusions

This population-based study provides the first comprehensive epidemiological profile of classic CAH in Hangzhou, China, over an 11-year period. The overall incidence is 3.24 per 100,000 live births, lower than many global estimates. Although temporal and regional differences were not statistically significant, the numerically higher incidence in urban areas and the predominance of salt-wasting cases highlight the need for improved diagnostic capacity in suburban and rural regions. Prenatal ultrasound findings, including NT thickening and enlarged adrenal glands, may facilitate earlier detection of at-risk fetus. The observed associations with CHD, CH, and cognitive developmental delay underscore the need for multidisciplinary, long-term management. Strengthening newborn screening programs, improving recall and referral systems, and enhancing front-line clinician education are essential to reducing the burden of this treatable disorder in Hangzhou and similar settings.

## Data Availability

The original contributions presented in the study are included in the article/supplementary material, further inquiries can be directed to the corresponding authors.

## References

[1] Luo P Li Q Yan B Xiong Y Li T Ding X. Prevalence, characteristics and risk factors of birth defects in central China livebirths, 2015–2022. Front Public Health. (2024) 12:1341378. 10.3389/fpubh.2024.1341378 39360259 PMC11445127

[2] Witchel SF. Congenital adrenal hyperplasia. J Pediatr Adolesc Gynecol. (2017) 30(5):520–34. 10.1016/j.jpag.2017.04.001 28450075 PMC5624825

[3] Speiser PW White PC. Congenital adrenal hyperplasia. N Engl J Med. (2003) 349(8):776–88. 10.1056/NEJMra021561 12930931

[4] Parsa AA New MI. Steroid 21-hydroxylase deficiency in congenital adrenal hyperplasia. J Steroid Biochem Mol Biol. (2017) 165(Pt A):2–11. 10.1016/j.jsbmb.2016.06.015 27380651

[5] Muthusamy K Elamin MB Smushkin G Murad MH Lampropulos JF Elamin KB. Clinical review: adult height in patients with congenital adrenal hyperplasia: a systematic review and metaanalysis. J Clin Endocrinol Metab. (2010) 95(9):4161–72. 10.1210/jc.2009-2616 20823467

[6] Speiser PW Arlt W Auchus RJ Baskin LS Conway GS Merke DP. Congenital adrenal hyperplasia due to steroid 21-hydroxylase deficiency: an endocrine society clinical practice guideline. J Clin Endocrinol Metab. (2018) 103(11):4043–88. 10.1210/jc.2018-01865 30272171 PMC6456929

[7] Baronio F Abrigo E Azzolini S Cavarzere P Cereda C De Sanctis L. Newborn screening for congenital adrenal hyperplasia due to 21 hydroxylase deficiency: the Italian experience 2006−2019. J Endocrinol Invest. (2025) 48(11):2713–20. 10.1007/s40618-025-02669-3 40952600

[8] Disorders ITFoIM. Newborn screening for congenital hypothyroidism and congenital adrenal hyperplasia. Indian J Pediatr. (2018) 85(11):935–40. 10.1007/s12098-018-2645-9 29549556

[9] Tsuji A Konishi K Hasegawa S Anazawa A Onishi T Ono M. Newborn screening for congenital adrenal hyperplasia in Tokyo, Japan from 1989 to 2013: a retrospective population-based study. BMC Pediatr. (2015) 15:209. 10.1186/s12887-015-0529-y 26671474 PMC4681082

[10] Li Z Huang L Du C Zhang C Zhang M Liang Y. Analysis of the screening results for congenital adrenal hyperplasia involving 7.85 million newborns in China: a systematic review and meta-analysis. Front Endocrinol (Lausanne). (2021) 12:624507. 10.3389/fendo.2021.624507 33967952 PMC8104032

[11] Screening Group of Neonatal Genetic Metabolic Disease SCoBDPaC, Chinese Preventive Medical Association. Consensus on diagnosis and treatment of inherited metabolic diseases by newborn screening. Chin J Neonatol. (2023) 38(07):385–94. 10.3760/cma.j.issn.2096-2932.2023.07.001

[12] Committee NHCCLCNGMDSI-lQA. Expert consensus on laboratory detection techniques for screening and diagnosis of congenital adrenal hyperplasia in newborns. Chinese Journal of Laboratory Medicine. (2019) 42(12):1014–9. 10.3760/cma.j.issn.1009-9158.2019.12.008

[13] Capital Institute of Pediatrics tCSGoNCotPGaDoCLH. Growth standard curves of birth weight, length and head circumference of Chinese newborns of different gestation. Chinese Journal of Pediatrics. (2020) 58(09):738–46. 10.3760/cma.j.cn112140-20200316-00242 32872714

[14] Huang H Cheng Y. Assessment of different neonatal birth weight references[J]. Chin. J. Reprod. Health. (2022) 33(2):130–34.

[15] Chu SY Tsai WY Chen LH Wei ML Chien YH Hwu WL. Neonatal screening for congenital adrenal hyperplasia in Taiwan: a pilot study. J Formos Med Assoc. (2002) 101(10):691–4. 12517042

[16] Wang X Lu X Zheng F Lin K Liao M Dong Y. Assessment of long-read sequencing-based congenital adrenal hyperplasia genotyping assay for newborns in fujian, China. Int J Neonatal Screen. (2025) 11(1):22. 10.3390/ijns11010022 40136637 PMC11942758

[17] Jia X Xie T Jiang X Tang F Tan M Chen Q. Neonatal screening for congenital adrenal hyperplasia in Guangzhou: 7 years of experience. Int J Neonatal Screen. (2025) 11(4):116. 10.3390/ijns11040116 41440812 PMC12733672

[18] Navarro-Zambrana AN Sheets LR. Ethnic and national differences in congenital adrenal hyperplasia incidence: a systematic review and meta-analysis. Horm Res Paediatr. (2023) 96(3):249–58. 10.1159/000526401 35973409

[19] Speiser P Chawla R Chen M Diaz-Thomas A Finlayson C Rutter M. Newborn screening protocols and positive predictive value for congenital adrenal hyperplasia vary across the United States. Int J Neonatal Screen. (2020) 6(2):37. 10.3390/ijns6020037 32832708 PMC7422998

[20] Chen Y Zhang H Zhang W Ning W Chen Y. The prevalence of hypospadias in newborn males in Hangzhou, China from 2011 to 2020: a cross-sectional population-based study. J Pediatr Urol. (2023) 19(5):583.e1–e7. 10.1016/j.jpurol.2023.06.009 37455207

[21] Varness TS Allen DB Hoffman GL. Newborn screening for congenital adrenal hyperplasia has reduced sensitivity in girls. J Pediatr. (2005) 147(4):493–8. 10.1016/j.jpeds.2005.04.035 16227036

[22] Guran T Yuruker E Anik A Atar M Camtosun E Eviz E. The first-year outcomes of the nationwide neonatal CAH screening in turkiye: high rate of false positives for 21-hydroxylase deficiency and a higher detection rate of non-classical cases. J Clin Res Pediatr Endocrinol. (2025) 17(4):488–93. 10.4274/jcrpe.galenos.2025.2024-9-11 40405612 PMC12699091

[23] Grosse SD Van Vliet G. How many deaths can be prevented by newborn screening for congenital adrenal hyperplasia? Horm Res. (2007) 67(6):284–91. 10.1159/000098400 17199092

[24] Odenwald B Nennstiel-Ratzel U Dorr HG Schmidt H Wildner M Bonfig W. Children with classic congenital adrenal hyperplasia experience salt loss and hypoglycemia: evaluation of adrenal crises during the first 6 years of life. Eur J Endocrinol. (2016) 174(2):177–86. 10.1530/EJE-15-0775 26563979

[25] Messina V Karlsson L Hirvikoski T Nordenstrom A Lajic S. Cognitive function of children and adolescents with congenital adrenal hyperplasia: importance of early diagnosis. J Clin Endocrinol Metab. (2020) 105(3):e683–91. 10.1210/clinem/dgaa016 31927590 PMC7343998

[26] Matarrelli B Khalil A Bernassola M Ranucci L Vizzielli G Lucidi A. Outcome of fetuses with early increased nuchal translucency: systematic review and meta-analysis. Ultrasound Obstet Gynecol. (2025) 66(2):147–54. 10.1002/uog.29260 40519157

[27] Sinajon P Chitayat D Roifman M Wasim S Carmona S Ryan G. Microarray and RASopathy-disorder testing in fetuses with increased nuchal translucency. Ultrasound Obstet Gynecol. (2020) 55(3):383–90. 10.1002/uog.20352 31115076

[28] Fincham J Pandya PP Yuksel B Loong YM Shah J. Increased first-trimester nuchal translucency as a prenatal manifestation of salt-wasting congenital adrenal hyperplasia. Ultrasound Obstet Gynecol. (2002) 20(4):392–4. 10.1046/j.1469-0705.2002.00803.x 12383325

[29] Masturzo B Hyett JA Kalache KD Rumsby G Jauniaux E Rodeck CH. Increased nuchal translucency as a prenatal manifestation of congenital adrenal hyperplasia. Prenat Diagn. (2001) 21(4):314–6. 10.1002/pd.54 11288125

[30] Haak MC Bartelings MM Jackson DG Webb S Van Vugt JM Gittenberger-de Groot AC. Increased nuchal translucency is associated with jugular lymphatic distension. Hum Reprod. (2002) 17(4):1086–92. 10.1093/humrep/17.4.1086 11925410

[31] Haak MC Van Vugt JM. Pathophysiology of increased nuchal translucency: a review of the literature. Hum Reprod Update. (2003) 9(2):175–84. 10.1093/humupd/dmg008 12751779

[32] Esser T Chaoui R. Enlarged adrenal glands as a prenatal marker of congenital adrenal hyperplasia: a report of two cases. Ultrasound in Obstetrics Gynecology. (2004) 23:293–7. 10.1002/uog.994 15027021

[33] Al-Alwan I Navarro O Daneman D Daneman A. Clinical utility of adrenal ultrasonography in the diagnosis of congenital adrenal hyperplasia. J Pediatr. (1999) 135(1):71–5. 10.1016/S0022-3476(99)70330-6 10393607

[34] Miles HL Gidlof S Nordenstrom A Ong KK Hughes IA. The role of androgens in fetal growth: observational study in two genetic models of disordered androgen signalling. Arch Dis Child Fetal Neonatal Ed. (2010) 95(6):F435–8. 10.1136/adc.2009.173575 20547585

[35] Melau C Nielsen JE Frederiksen H Kilcoyne K Perlman S Lundvall L. Characterization of human adrenal steroidogenesis during fetal development. J Clin Endocrinol Metab. (2019) 104(5):1802–12. 10.1210/jc.2018-01759 30590593 PMC6456011

[36] Lingaiah K Parshwanath BA Mysore SR Krishnamurthy B Ramachandra NB. A rare case of congenital heart disease with ambiguous genitalia. Indian J Hum Genet. (2010) 16(3):166–8. 10.4103/0971-6866.73414 21206707 PMC3009430

[37] Thorat JV Tambolkar S. Persistent urogenital sinus leading to hydrometrocolpos in a female child with features of McKusick-kaufman syndrome. Cureus. (2024) 16(6):e61957. 10.7759/cureus.61957 38978907 PMC11230620

[38] Alzanbagi MA Milyani AA Al-Agha AE. Growth characteristics in children with congenital adrenal hyperplasia. Saudi Med J. (2018) 39(7):674–8. 10.15537/smj.2018.7.22193 29968889 PMC6146245

[39] Improda N Ponmani C Schoenmakers N Senniappan S Atterbury A Barnicoat A. Intrafamilial phenotypic variability and consequences of non-compliance with treatment in congenital adrenal hyperplasia and congenital hypothyroidism within a single family. Horm Res Paediatr. (2017) 88(2):172–8. 10.1159/000465521 28359061

[40] Dörr HG Penger T Albrecht A Marx M Völkl TMK. Birth size in neonates with congenital adrenal hyperplasia due to 21-hydroxylase deficiency. J Clin Res Pediatr Endocrinol. (2019) 11(1):41–5. 10.4274/jcrpe.galenos.2018.2018.0149 PMC6398197 30178749

[41] Bonfig W Schmidt H Schwarz HP. Growth patterns in the first three years of life in children with classical congenital adrenal hyperplasia diagnosed by newborn screening and treated with low doses of hydrocortisone. Horm Res Paediatr. (2011) 75(1):32–7. 10.1159/000316973 20714115

[42] Perry R Kecha O Paquette J Huot C Van Vliet G Deal C. Primary adrenal insufficiency in children: twenty years experience at the sainte-justine hospital, Montreal. J Clin Endocrinol Metab. (2005) 90(6):3243–50. 10.1210/jc.2004-0016 15811934

[43] Amais DSR Da Silva TER Barros BA De Andrade JGR De Lemos-Marini SHV De Mello MP. Sex dimorphism of weight and length at birth: evidence based on disorders of sex development. Ann Hum Biol. (2022) 49(7-8):274–9. 10.1080/03014460.2022.2134452 36218438

[44] Webb EA Elliott L Carlin D Wilson M Hall K Netherton J. Quantitative brain MRI in congenital adrenal hyperplasia: *in vivo* assessment of the cognitive and structural impact of steroid hormones. J Clin Endocrinol Metab. (2018) 103(4):1330–41. 10.1210/jc.2017-01481 29165577 PMC6018658

[45] Kong L Chen X Gissler M Lavebratt C. Relationship of prenatal maternal obesity and diabetes to offspring neurodevelopmental and psychiatric disorders: a narrative review. Int J Obes (Lond). (2020) 44(10):1981–2000. 10.1038/s41366-020-0609-4 32494038 PMC7508672

